# Artificial Intelligence for Microbial Isolation and Cultivation: Progress and Challenges

**DOI:** 10.3390/microorganisms14030654

**Published:** 2026-03-13

**Authors:** Mingyu Li, Xiangwu Yao, Meng Zhang, Baolan Hu

**Affiliations:** 1 State Key Laboratory of Soil Pollution Control and Safety, College of Environmental and Resource Sciences, Zhejiang University, Hangzhou 310058, China; leethebeauty@zju.edu.cn (M.L.); xwyao@zju.edu.cn (X.Y.); zhangm_environment@zju.edu.cn (M.Z.); 2 College of Environment and Resource Sciences, Zhejiang University, Hangzhou 310058, China; 3 Zhejiang Key Laboratory of Water Pollution Control and Water Ecological Health, Hangzhou 310058, China

**Keywords:** microbial isolation and cultivation, artificial intelligence, machine learning, deep learning, microbial resource discovery, culturomics

## Abstract

Microbial resources are crucial for biotechnology development and fundamental scientific research. Traditional microbial techniques fail to isolate and cultivate the vast majority of microorganisms in nature, severely limiting the discovery of novel microbial resources. The rise in artificial intelligence (AI) technologies provides new computational tools to overcome bottlenecks in microbial resource discovery and utilization. This review comprehensively examines the development of AI technologies in microbial isolation and cultivation over the past three decades from the perspective of microbial resource discovery. We propose a five-stage framework: the germination period (1997–2008), the early exploration period (2008–2015), the rapid development period (2015–2019), the deep learning (DL) explosion period (2020–2022), and the AI integration period (2023–present). We focus on how AI technologies at each stage address core challenges in microbiology—including insufficient knowledge reserves, dynamic phenotypic changes, and complex cultivation conditions—through applications at the genome, individual, and community levels. Our analysis demonstrates that, as AI technologies advance iteratively, microbial isolation and cultivation methods are transitioning from experience-driven to data-driven approaches, from single-objective to systematic integration, and from passive screening to active design. This methodological transition is expanding the scope of microbial resource discovery.

## 1. Introduction

Microorganisms represent the most widely distributed and species-diverse life forms on Earth. It is estimated that the total number of microbial species on Earth reaches approximately 10^12^ [1], yet less than 1% have been successfully isolated and cultivated to date [2]. These uncultured microorganisms, referred to as “microbial dark matter,” harbor rich genetic information and diverse metabolic functions of tremendous research value. The core reasons for this predicament include: (1) severely insufficient understanding of the physiological and ecological requirements of most microorganisms; (2) high dynamicity of microbial growth phenotypes influenced by environmental factors, making real-time tracking difficult with traditional methods; (3) inadequate elucidation of complex inter- and intra-species microbial interaction mechanisms; and (4) the vast parameter space of cultivation conditions, rendering traditional trial-and-error screening strategies highly inefficient. Recent advances in culturomics [3] and high-throughput cultivation platforms [4] have begun to address these bottlenecks, yet systematic strategies for efficiently exploring microbial dark matter remain limited.

AI technologies, particularly machine learning (ML) and DL, offer novel tools for addressing these challenges. Compared to traditional approaches, AI offers three core advantages: first, mining hidden patterns from massive, heterogeneous multi-source data to fill gaps in human knowledge; second, real-time processing of high-dimensional dynamic data to capture rapid changes in microbial growth precisely; and third, system-level optimization capabilities to search for optimal solutions in complex parameter spaces efficiently. Since the first application of AI to microbial cultivation optimization in the late 1990s [5,6], AI technologies have undergone nearly three decades of development, establishing a comprehensive technical framework encompassing genome analysis, individual recognition, and community analysis.

Before presenting the framework, it is essential to clarify the scope and key definitions used in this review. Microbial isolation refers to the process of separating individual microbial strains from complex environmental or clinical samples, typically involving sample processing, selective enrichment, physical separation (e.g., colony picking, single-cell sorting, microfluidic droplet encapsulation), and taxonomic identification. Microbial cultivation is the process of maintaining and propagating isolated strains under controlled laboratory conditions, encompassing medium design, optimization of environmental parameters (temperature, pH, oxygen level, incubation time), and growth monitoring. While these two processes are sequential in practice, the AI technologies discussed in this review often contribute to both. For instance, genomic prediction of nutritional requirements informs both the selective enrichment step in isolation and the medium optimization step in cultivation. Throughout the text, we evaluate AI contributions using the following key metrics where applicable: isolation success rate, culturability (percentage of species successfully grown), time-to-isolation, identification accuracy, and reproducibility across protocols and laboratories. The term “unculturable” is used operationally to denote microorganisms that have not yet been cultivated under the conditions tested in the laboratory, rather than implying an absolute biological limitation.

This review divides the development history into five stages: the germination period, the early exploration period, the rapid development period, the DL explosion period, and the AI integration period. We comprehensively review the evolution of AI applications in microbial isolation and cultivation across three biological levels—genome, individual, and community—and evaluate the effectiveness of technological iterations in addressing key challenges, including insufficient microbial knowledge, rapid dynamic changes, and cultivation condition optimization.

The five-stage framework is defined by three objective criteria applied to each period: (i) the dominant model family—ranging from classical neural networks and shallow ML (Stages 1–2), to deep convolutional and recurrent architectures (Stage 3), advanced architectures including reinforcement learning and neural ODEs (Stage 4), and multi-modal foundation-model-scale systems (Stage 5); (ii) the primary data modality—from tabular process data and fatty-acid profiles (Stage 1), to genomic sequences and gene-expression matrices (Stage 2), image and spectral data (Stage 3), multi-omics integration (Stage 4), and cross-modal fusion of genomics, imaging, spectroscopy, and literature knowledge (Stage 5); and (iii) the level of automation—from offline, human-in-the-loop decision support (Stages 1–3), to semi-automated closed-loop optimization (Stage 4), and fully autonomous experimental design and execution (Stage 5). These criteria are discussed at the opening of each stage section below, and representative technologies illustrating each transition are listed in Table 1.

## 2. Germination Period (1997–2008): From Bioprocess Control to Microbial Cultivation Optimization

The germination period represents the initial exploration phase when AI technologies were first applied to microbial cultivation. The core characteristic of this stage was the integration of control theory with neural networks to address state estimation and condition-optimization problems in microbial fermentation processes. The main challenge facing the microbiology community at this time was the low efficiency of traditional experience-driven cultivation methods and the difficulty in establishing precise mathematical models due to noise and uncertainty in biological processes.

In 1997, Patnaik pioneered the application of hybrid neural networks to the control of the recombinant Escherichia coli fermentation process [5], marking the formal entry of AI technologies into the field of microbial cultivation. This study first demonstrated that, even with incomplete cultivation environment information, AI could accurately assess microbial growth states by learning from historical data. Between 2001 and 2007, Patnaik further developed hybrid neural network methods for simulating fed-batch fermentation [6] and systematically explored intelligent approaches for optimizing poly-β-hydroxybutyrate (PHB) production, noise modulation, and cultivation control [7,8,9]. The 2007 study [9] compared neural network and cybernetic models of PHB biosynthesis in Ralstonia eutropha, revealing a trade-off between “predictive performance” and “interpretability”—an issue that remains a research focus today.

In species identification, Slabbinck et al. (2008) [10] used artificial neural networks for genus-wide species-level identification of Bacillus. Based on fatty acid methyl ester gas chromatography analysis, this study trained multilayer neural network models using 1071 fatty acid profile data, achieving species-level identification accuracy of 85–90%, significantly outperforming traditional pattern matching methods (approximately 60–70%). Accurate and rapid species identification enables early recognition of known strains during isolation, allowing resources to be concentrated on cultivating novel strains, and provides efficient tools for establishing large-scale strain libraries.

The technologies during the germination period primarily focused on single-strain cultivation optimization, without using genomic information or conducting community-level analysis. Significant limitations included: insufficient data-acquisition capabilities, particularly the near absence of data on difficult-to-cultivate microorganisms; computational constraints limiting the scale of neural networks; and an application scope restricted to known cultivable microorganisms. These limitations pointed to directions for the next stage: utilizing genomic information to predict cultivation requirements, developing high-throughput screening technologies, and studying microbial community interactions. Despite these limitations, the germination period established a critical proof of concept: AI-optimized fermentation parameters were directly adopted in industrial PHB production processes, demonstrating tangible experimental impact. The maturation of high-throughput sequencing technologies and the rise in metagenomics provided the technical foundation for subsequent developments.

## 3. Early Exploration Period (2008–2015): Systematic Application of Traditional ML

The early exploration period witnessed the systematic application of traditional ML methods in microbiology. This stage coincided with the maturation of high-throughput sequencing technologies and the rise in metagenomics, providing abundant data resources for ML. Researchers began systematically introducing algorithms such as support vector machines, random forests, and extreme learning machines, transforming research methods from “rule-based” to “data-driven” paradigms.

At the genome level, preliminary progress was achieved in metagenomic data analysis. Metagenomics technology enables the study of difficult-to-cultivate microorganisms by directly extracting and sequencing DNA from environmental samples without prior cultivation [58]. By bypassing cultivation entirely, metagenomics provides access to the genomic content of the estimated > 99% of environmental microorganisms that resist laboratory cultivation [2], thereby generating raw data from which AI models can infer metabolic capabilities and predict cultivation requirements. In 2012, Rasheed and Rangwala [11] used extreme learning machines for metagenomic sequence classification, demonstrating excellent performance in training speed and short sequence prediction. This technology enables rapid identification of potentially novel microbial taxa from complex environmental samples and preliminary assessment of their cultivability based on genomic features. For example, by identifying complete essential amino acid synthesis pathways, one can infer that the microorganism may require complex organic nutrient sources; by discovering genes for remarkable cofactor synthesis, one can suggest trace elements to be added to the culture medium. This “sequence first, predict second, cultivate third” strategy represents an essential shift in the paradigm of microbial resource discovery.

At the individual and community levels, applications during this period were relatively limited, primarily to accumulate data and experience for subsequent breakthroughs in DL. Traditional ML methods achieved good results on specific tasks, but shallow model architectures struggled to capture highly nonlinear, complex relationships. Significant limitations included: feature engineering requiring extensive domain knowledge and manual design, limiting model generalization capability; difficulty in effectively integrating data from different sequencing platforms and experimental conditions; and limited high-quality annotated data resources, with extremely scarce data for rare species. These limitations prompted researchers to begin exploring DL methods after 2015. The breakthrough of the AlexNet deep neural network in image recognition in 2012, and the popularization of graphics processing unit (GPU) parallel computing technology, provided the technical foundation for DL applications in microbiology.

## 4. Rapid Development Period (2015–2019): Initial Application of DL

The rapid development period marks the stage when DL technologies began to emerge in microbiology. DL achieved breakthroughs in gene expression data analysis and image recognition, demonstrating its potential for application in microbiological research. Traditional ML methods continued to be widely applied and refined, complementing DL approaches.

At the genome level, Tan et al. (2016) [12] first applied denoising autoencoders to integrate Pseudomonas aeruginosa gene expression data, proposing the ADAGE method. This study integrated over 900 publicly available gene expression datasets, using unsupervised learning to automatically learn biologically relevant patterns from massive data, successfully identifying gene modules closely related to microbe-host interactions and discovering new virulence factors and metabolic pathway mechanisms.

At the community level, Bokulich et al. (2016) [13] employed ML methods to analyze correlations among wine microbiome composition, metabolome characteristics, and fermentation behavior. By longitudinally tracking over 200 commercial wine fermentation samples, they found significant correlations between microbial community composition and wine chemical composition.

In rapid identification, Andini et al. (2017) [14] developed a DNA melting curve-based microbial typing method using naive Bayes classifiers to classify high-resolution melting analysis data, effectively distinguishing closely related bacterial species with classification accuracy exceeding 90% and detection time reduced to within 2 h. Marques et al. (2017) [15] applied hybrid modeling methods in Alcaligenes extracellular polysaccharide production, achieving average prediction errors of 5–8%, outperforming pure mechanistic models (15–25%) and pure neural network models (8–12%).

At the individual level, DL significantly improved the automation of microbial image analysis. DiMucci et al. (2018) [16] applied random forests to microbial interaction networks, demonstrating that only 5% of known interactions were sufficient to predict the remaining interactions with 80% accuracy, suggesting the possibility of reducing experimental workload. This technology can predict which “helper strains” difficult-to-cultivate microorganisms depend on for nutrients or signaling molecules, helping to design rational co-cultivation systems and improving isolation success rates for these organisms.

Ancin-Murguzur et al. (2018) [17] developed an automated image analysis tool based on support vector machines, reducing manual workload by over 90%. Bellenberg et al. [18] developed a deep neural network system for automated microscopic analysis of acidophilic microorganism colonization on metal sulfides, achieving processing speeds over 100 times faster than manual analysis with accuracy exceeding 95%. These technologies enable rapid and accurate identification and counting of colonies with different morphologies from mixed culture plates, achieving high-throughput initial screening. Czajka et al. (2018) [19] used ML-assisted engineering to modify Yarrowia lipolytica for β-ionone production, improving yields by over 10-fold.

The rapid development period demonstrated the application potential of DL in specific areas of microbiology. However, applications remained relatively limited, primarily concentrated in image analysis and gene expression data analysis. The “black box” nature of DL models was questioned in biological research, and interdisciplinary talents remained relatively scarce. Notably, the hybrid modeling approach of Marques et al. [15] was experimentally validated in pilot-scale bioreactors, and the interaction predictions of DiMucci et al. [16] informed the design of co-cultivation experiments that successfully recovered previously uncultured species. These factors drove the development of more advanced DL models and algorithms.

## 5. DL Explosion Period (2020–2022): Completion of Multi-Level Technical Framework

The DL explosion period was a critical time when DL technologies flourished across microbial isolation and cultivation. Advanced model architectures, such as deep reinforcement learning and neural ordinary differential equations, have made breakthroughs in microbial process control and metabolic prediction. The COVID-19 pandemic accelerated the application of AI technologies in pathogen detection and drug discovery. DL began to form a comprehensive technical framework covering the genome, individual, and community levels.

### 5.1. Genome Level

In 2020, Ren et al. [20] proposed DeepVirFinder, a reference-free, alignment-free DL method for identifying viral sequences from metagenomic data. Using deep convolutional neural networks to automatically learn features from DNA sequences without relying on known virus sequence databases, it achieved comprehensive identification accuracy of 93–98% across different sequence lengths (300 to 3000 base pairs), outperforming existing methods. This breakthrough eliminated dependence on reference databases, enabling the discovery of entirely new types of microorganisms and providing a theoretical basis for phage-mediated microbial cultivation strategies. Taylan et al. (2020) [21] used ML to optimize the asymmetric reduction in acetophenone catalyzed by whole cells of Lactobacillus, thereby improving yields by over 40% and achieving enantioselectivity exceeding 95%.

### 5.2. Community Level

Interpretable AI and DL made essential advances in microbiome analysis. Lee et al. (2020) [22] used DL to predict microbial interaction relationships from self-organized spatiotemporal patterns, achieving 84% fit with actual observations and effectively distinguishing among different types of interactions (competition, symbiosis, predation, etc.). Prifti et al. (2020) [23] proposed the “predomics” method, customized for metagenomic data analysis with particular emphasis on model interpretability, achieving prediction accuracy at least comparable to that of state-of-the-art methods across over 100 datasets.

### 5.3. Individual Level

Deep reinforcement learning achieved breakthroughs in microbial process control. Treloar et al. (2020) [24] first demonstrated the effectiveness of deep reinforcement learning in microbial co-culture control. Using deep Q-networks (an algorithm combining DL with reinforcement learning) to control multi-species abundance in continuous cultivation systems, the intelligent control system effectively maintained multi-species abundance at target levels, significantly outperforming traditional proportional-integral controllers under low-sampling-frequency conditions. More importantly, reinforcement learning methods do not require establishing precise system dynamics models. This “learn while cultivating” strategy is particularly suitable for slow-growing, physiologically complex, difficult-to-cultivate microorganisms, with systems dynamically adjusting strategies to respond to changes in microbial state.

The DL explosion period achieved multi-level application of AI technologies in microbial isolation and cultivation but still faced challenges: level fragmentation—technologies at different levels were relatively independent, with genomic predictions unable to guide actual isolation operations directly, individual recognition technologies failing to utilize genomic information thoroughly, and community analysis results difficult to translate into actionable cultivation strategies; strong data dependency, primarily relying on supervised learning; limited generalization capability; and insufficient interpretability. Among the methods discussed, deep reinforcement learning [24] was experimentally validated in real bioreactor settings, and the DeepVirFinder tool [20] has been widely adopted in environmental viromics studies, collectively demonstrating that these AI advances translate into measurable experimental outcomes. These limitations clearly indicated directions for the next stage: developing end-to-end integrated systems and exploring transfer learning and active learning methods.

## 6. AI Integration Period (2023–Present): Maturation of End-to-End Intelligent Systems

The core characteristics of the AI integration period are multi-level technology integration, cross-domain knowledge fusion, and the transition from data-driven to knowledge-guided approaches. Cutting-edge technologies such as transfer learning and multimodal fusion have been introduced to the field of microbiology. Active learning and reinforcement learning have enabled microbial cultivation systems to optimize autonomously. A key development is the emergence of end-to-end intelligent systems—full-process intelligence from genomic prediction to automated cultivation to pure culture acquisition—driving methodological transitions from “analyzing the known” to “discovering the unknown” and from “passive screening” to “active design.”

### 6.1. Genome Level

DL significantly enhanced metagenomic analysis capabilities and the prediction of cultivation conditions. Between 2021 and 2022, Arisdakessian et al. [25], Bai et al. [26], and Zhang et al. [27] developed multiple DL-based metagenomic analysis tools that automate viral metagenomic binning, bacteriophage genome identification, and metagenomic identification. In 2023, Bi et al. [28] developed etiBsu1209, a comprehensive multiscale metabolic model for Bacillus subtilis that enables multiscale metabolic predictions to guide cultivation strategies. Ultra-deep metagenomic analysis has also revealed resistome characteristics in pristine environments. In 2025, Zhao et al. [29] conducted systematic metagenomic sequencing (1.8 Tb data) of pristine saline lakes on the Qinghai–Tibet Plateau, detecting 756 antibiotic resistance gene subtypes and identifying the clinically relevant polymyxin resistance gene ugd as the most abundant, with 183 horizontal gene transfer events across 18 genera.

In gene function annotation, DL technologies have significantly improved the efficiency of annotating microbial genomes. In 2026, Palsson and Lee et al. [30] systematically summarized AI applications in microbial gene function discovery, including DL tools such as DeepTFactor for transcription factor prediction and DeepECtransformer for enzyme commission (EC) number prediction. These tools, combined with experimental validation methods such as chromatin immunoprecipitation sequencing, have increased the number of verified transcription factors in E. coli from 242 to 276 and can predict approximately 5000 enzyme functional classifications, providing computational tools for the systematic analysis of microbial metabolic capabilities. Notably, this study emphasized the importance of interpretable AI, where attention-weight analysis can identify sequence motifs related to enzyme function, providing a basis for understanding the molecular mechanisms underlying predictions.

In large-scale genomic data search, Shen et al. (2024) [31] developed the LexicMap algorithm for rapid “gold-standard” alignment against millions of microbial genomes. This tool is 72 times faster than the traditional sequence alignment tool BLASTn (https://blast.ncbi.nlm.nih.gov/Blast.cgi; accessed 4 February 2026) and 872 times faster than the next-generation alignment tool MMSeqs2 (https://github.com/soedinglab/MMseqs2; accessed 4 February 2026), requiring only 7 GB of memory (traditional methods require over 300 GB), enabling rapid searching for specific genes in the AllTheBacteria database (containing 2.3 million bacterial and archaeal genomes). This provides efficient tools for epidemiological investigations tracking the spread of resistance genes and ecological studies analyzing the global distribution of genes.

A notable advance came from the carbon and nitrogen source preference prediction model developed by Wang et al. (2025) [32]. Based on protein sequence features, this model uses random forests to predict microbial utilization of 214 carbon sources and 95 nitrogen sources, achieving 87.2% accuracy. This study enables the design of personalized culture media for uncultured microorganisms without experimentation, demonstrating that direct prediction from genomic sequences to cultivation conditions is feasible. For microorganisms whose genome sequences have been obtained through metagenomics but have never been cultivated, nutritional requirements can be directly predicted, thereby significantly reducing the need for trial-and-error experimentation.

### 6.2. Individual Level

#### 6.2.1. Colony Detection

In 2023, Yang et al. [33] developed a microbial colony detection system based on YOLOv8 (an efficient real-time object detection network), achieving average colony recognition accuracy exceeding 93% at real-time speeds. In 2025, Majchrowska et al. [34] released the AGAR image dataset containing 18,000 high-resolution images with 336,442 colonies annotated by professional microbiologists with over 20 years of experience, representing the largest publicly available dataset and providing a benchmark for standardized algorithm evaluation.

#### 6.2.2. Single-Cell Rapid Identification

The combination of Raman spectroscopy and DL has achieved truly “cultivation-free, extraction-free” rapid identification. In 2023, Lyu et al. [35] developed a rapid prediction system for multidrug-resistant Klebsiella pneumoniae based on DL analysis of surface-enhanced Raman spectroscopy (SERS), achieving accuracy exceeding 95% with a detection time of under 2 h. Liu et al. [36] introduced the Transformer architecture (a DL model based on attention mechanisms) into Raman spectroscopy classification, developing a deep-sea cold seep bacteria classification system with 95.3% accuracy, capable of single-cell-level identification and with detection time reduced to under 5 min. In 2025, Lü et al. [37] developed a DL model for Raman spectroscopy that can identify 27 classes of environmental microorganisms without cultivation, achieving 89.3% accuracy and 100 times faster than traditional sequencing. Zheng et al. (2025) [38] developed DL-enhanced hyperspectral imaging technology for rapid screening of microplastic-cometabolizing bacteria, achieving 89.7% accuracy and a 50-fold increase in throughput. Bi et al. (2025) [39] developed a paper-based SERS chip combined with an adaptive attention neural network, achieving pathogen detection within 5 min at a cost of less than 1 yuan with accuracy exceeding 95%. Single-cell-level rapid identification enables direct identification and sorting of target cells from mixed communities, combined with microfluidic technology to achieve actual single-cell isolation cultivation, significantly improving detection efficiency for rare strains. It should be noted, however, that identification accuracy alone does not guarantee successful isolation or cultivation. The outputs of spectroscopic identification serve as an upstream selection step: once target cells are identified, they must be physically separated (e.g., via optical tweezers, fluorescence-activated cell sorting (FACS), or microfluidic sorting), transferred to appropriate culture media whose composition may be informed by genomic predictions [32], and incubated under optimized conditions. Each of these downstream steps introduces additional failure modes. Consequently, reported identification accuracies (89–95%) should be interpreted as upper bounds on the initial screening step, not as overall isolation success rates.

Ul Ain and Asif (2024) [40] developed deep transfer learning combined with hyperspectral technology for the accurate identification of foodborne bacteria, achieving multimodal fusion and rapid, non-destructive detection. Asama et al. (2024) [41] developed a droplet microfluidic platform combined with ML for high-throughput detection of G-protein-coupled receptor (GPCR) agonist peptides expressed in yeast. Potenza et al. (2025) [42] developed an induced-droplet ovalisation image-based microfluidic method for high-throughput, label-free characterization of microbial proteolytic strains from wastewater sludge.

#### 6.2.3. Autonomous Learning Systems

The BacterAI system published by Dama et al. (2023) [43] and the high-throughput microbial culturomics platform developed by Huang et al. [44] represent notable advances. The BacterAI system, without prior knowledge, autonomously completes experimental design, execution, and result analysis via active learning, successfully mapping microbial metabolic profiles. Compared to a random experimental design, the BacterAI system reduces the number of experimental iterations by approximately 50% while achieving the same level of knowledge coverage and discovering multiple metabolic capabilities in E. coli and Pseudomonas not predicted by genome annotation. Huang et al.’s platform system combines liquid handling robots, automated imaging systems, and ML algorithms to achieve end-to-end automation from sample processing to strain identification, improving processing throughput by over 100-fold, isolating and cultivating over 10,000 bacterial strains from the human gut microbiome, and discovering multiple new species and previously uncultured strains.

In 2025, Liu et al.’s MediaMatch platform [45] employed XGBoost (an efficient gradient boosting decision tree algorithm) to integrate large-scale culture medium formulation data, constructing a database covering over 5000 medium formulations and 20,000 microbial growth records. The platform successfully cultivated 38% of species classified as “unculturable” in databases, improving medium design efficiency by 40-fold. Feature importance analysis revealed that nitrogen source type, trace element ratios, and pH-buffering systems are the three key factors affecting cultivation success rates.

A representative case demonstrating long-term cultivation strategies is the isolation of Candidatus Methylomirabilis sinica, a nitrate-dependent anaerobic methanotroph. In 2024, Yao et al. [46] successfully obtained the first pure culture of this bacterium through over 1000 days of enrichment cultivation combined with single-cell sorting and gradient dilution, confirming its independent catalysis of complete denitrification-coupled anaerobic methane oxidation.

Beyond isolation, metabolic regulation of cultivated microorganisms enables functional optimization. Yao and Hu (2024) [47] further proposed that Methylomirabilis bacteria could serve as indirect N_2_O sinks, offering potential for simultaneous CH_4_ and N_2_O mitigation. Yao et al. (2025) [48] demonstrated that iron acts as a metabolic “switch” in M. sinica, with low iron (20 μM) promoting growth, while high iron (40 μM) enhances metabolic activity by reprogramming carbon metabolism from the Calvin cycle to the serine pathway.

### 6.3. Community Level

Neural ordinary differential equations (models combining differential equations with neural networks) and graph neural networks achieved breakthroughs in microbiome analysis. Wang et al. (2023) [49] proposed the mNODE model, which applies neural ordinary differential equation (ODE) methods to predict microbiome metabolic profiles, achieving a balance between high performance and interpretability. On human gut microbiome data, the metabolic profile prediction fit reached 75%, outperforming random forest methods (62%) and standard neural network methods (68%), and the model parameters have clear biological meaning, interpretable as interaction strengths between microorganisms and metabolites. The mNODE model can predict dynamic changes in microbial communities under different cultivation conditions, guiding adjustments to cultivation strategies. Understanding inter-bacterial mutualism is crucial for the design of co-cultivation systems. Zhao et al. (2023) [50] investigated bacterial mutualism in composting systems through high-throughput sequencing and over 3000 bacterial pair co-cultivation experiments, discovering that high-temperature stress selectively enriches slow-growing species that promote other bacteria by sharing cobalamin, with mutualistic relationships accounting for 31–45% of interactions.

Graph neural networks (DL models capable of processing graph-structured data) made important advances in predicting microbe-disease associations and microbial interactions. Wang et al. [51] achieved prediction of microbial-disease associations by learning global graph features from heterogeneous networks. Pan et al. (2023) [52] developed a model for predicting bacteriophage-host interactions with comprehensive prediction accuracy exceeding 92% across multiple datasets. Wang et al. (2024) [53] proposed multi-source feature fusion methods to identify keystone species in microbial communities, guiding targeted isolation strategies. Wang et al. (2025) [54] developed a graph convolutional attention network to identify associations between microbes and diseases, which can accurately predict diseases such as cirrhosis and epilepsy. These technologies can identify key microorganisms associated with specific functions within complex communities, predict inter-microbial interactions, and provide a basis for designing co-cultivation systems.

Wu et al. (2024) [55] proposed a data-driven method for predicting colonization outcomes of exogenous microorganisms in complex communities without requiring kinetic models. Mermans et al. (2025) [56] utilized flow cytometry combined with quantitative analysis methods to quantify synthetic community composition, showing promising results in simulated communities and providing new tools for community construction. Bartsch et al. (2025) [57] combined anaerobic incubator arrays, automated colony-picking robots, nanopore sequencing, and machine-learning colony recognition algorithms to propose the multidimensional culturomics concept, thereby improving the proportion of cultivable microorganisms from the traditional baseline of less than 1% [2] to 15–30%, representing a 15- to 30-fold increase attributable to the integration of automated colony selection, real-time nanopore-based taxonomic identification, and machine-learning-guided iterative medium optimization.

### 6.4. Summary

**Figure 1:**
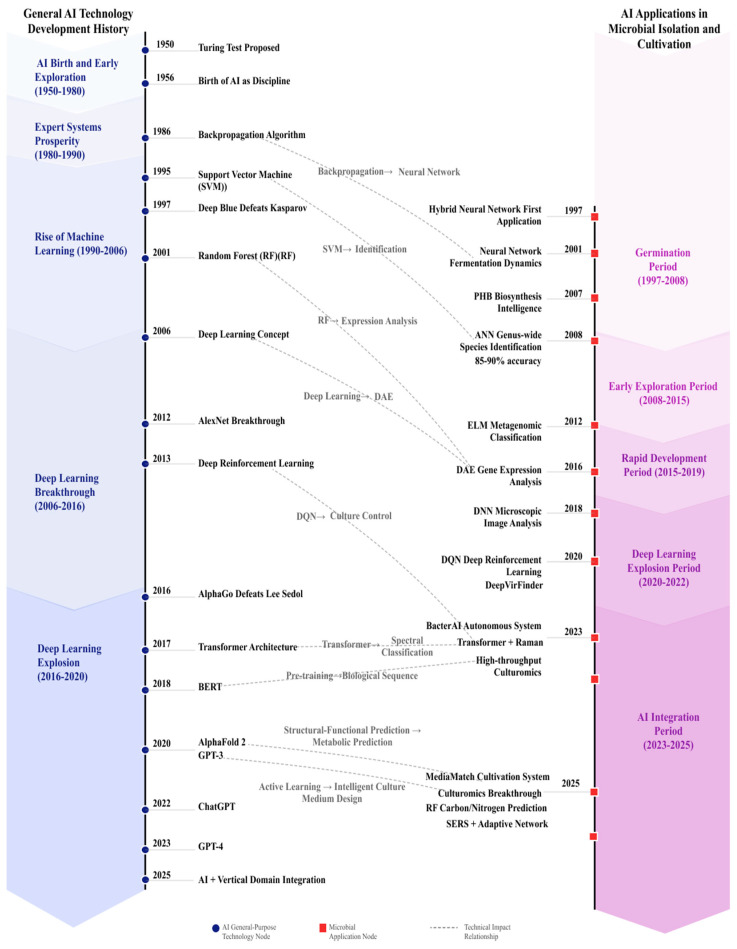
Dual Timeline Comparison of AI Technology Development and Microbial Isolation/Cultivation Applications. The upper timeline illustrates key milestones in general AI development (from classical neural networks to large language models), while the lower timeline maps corresponding applications in microbial isolation and cultivation. The five colored stages correspond to the developmental periods defined in this review: germination (1997–2008), early exploration (2008–2015), rapid development (2015–2019), DL explosion (2020–2022), and AI integration (2023–present). Arrows indicate direct technology transfer events between advances in general AI and microbiological applications.

The AI integration period achieved comprehensive maturation of AI technologies in microbial isolation and cultivation (Figure 1). Landmark breakthroughs include: end-to-end intelligence—defined here as automated pipelines spanning from genomic prediction through physical isolation to culture verification, though human oversight remains necessary for experimental design validation and quality control [44,55], autonomous learning capability [43], significantly improved culturability (MediaMatch successfully cultivating 38% of “unculturable” species [45], culturomics improving culturability to 15–30% [57]), single-cell resolution [35,36,37,39], enhanced interpretability [23,30,49] and the physiological and biochemical studies of specific microorganisms [46,47,48]. Critically, several of these advances have demonstrated real-world experimental impact: MediaMatch [45] predictions were validated by successfully cultivating 38% of previously uncultured species in laboratory experiments, and the BacterAI system [43] autonomously discovered metabolic capabilities not predicted by genome annotation through actual cultivation trials. However, this stage still faces challenges: high-quality cultivation data and annotated data remain scarce, with insufficient data standardization; model generalization across laboratories and environments still needs improvement; the “black box” nature of DL models persists; experimental validation costs and cycles remain limiting factors; and computational resources, automation equipment, and professional talent costs limit technology adoption. Figure 1 illustrates the dual timeline of general AI milestones and their corresponding applications in microbial isolation and cultivation across the five stages.

## 7. Discussion

### 7.1. Core Challenges

**Figure 2:**
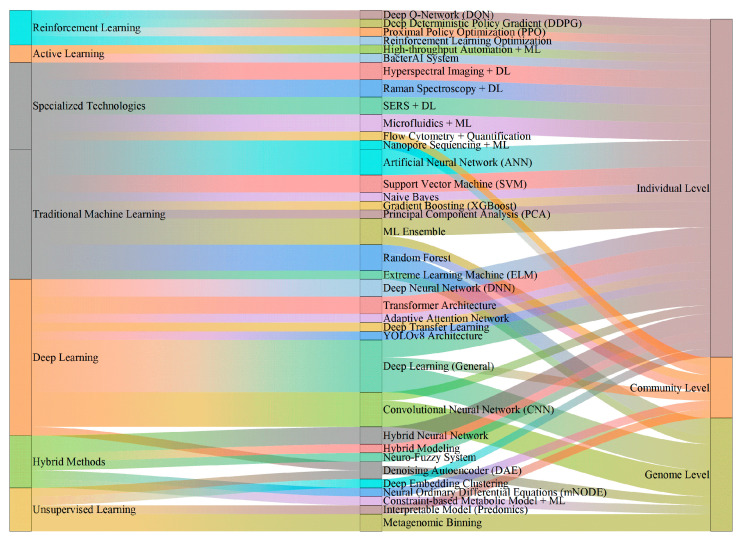
Three-Level Framework of AI Applications in Microbiology: Genome, Individual, and Community. The framework organizes AI contributions across three biological levels. At the genome level, AI enables metagenomic analysis, gene function annotation, and prediction of cultivation conditions. At the individual level, AI drives colony detection, single-cell identification, and autonomous cultivation optimization. At the community level, AI supports interaction prediction, microbiome modeling, and co-cultivation design. Bidirectional arrows indicate information flow between levels (e.g., genomic predictions informing individual-level medium design).

The core challenge in microbial resource discovery lies in the “enormous uncultured microbial diversity” [2], stemming from “insufficient knowledge” of microbial physiological and ecological requirements, “rapid changes” in microbial phenotypes, and the “vast parameter space” of cultivation conditions. AI technologies have provided systematic computational approaches to these core challenges over the past three decades (Figure 2): inferring potential metabolic capabilities and nutritional requirements from the genome level through metagenomic analysis [20,25,26,27], metabolic network prediction, and carbon-nitrogen source preference prediction [32]; achieving adaptive cultivation control through deep reinforcement learning [24] for real-time monitoring and dynamic adjustment of cultivation conditions; and efficiently exploring the vast cultivation condition parameter space through active learning strategies [43] and intelligent medium prediction systems [45].

However, AI technologies have also revealed their own limitations while addressing these problems: for truly rare or novel microorganisms, high-quality cultivation data and annotated data remain extremely scarce; most DL models remain “black boxes” with insufficient interpretability; experimental validation costs and cycles remain limiting factors; although autonomous experimental systems are taking shape, achieving fully autonomous processes still requires overcoming multiple challenges; and computational resources, automation equipment, and professional talent costs limit technology adoption. The environmental footprint of AI technologies themselves warrants consideration. Yu et al. (2024) [59] estimated that the top 20 AI systems could collectively emit up to 102.6 Mt CO_2_ equivalent annually, suggesting that future AI applications in microbiology should balance technological advancement with sustainable development.

### 7.2. Benchmarking and Reproducibility Challenges

A critical barrier to evaluating progress in AI-assisted microbial isolation and cultivation is the lack of standardized benchmarks and reporting practices—several specific challenges merit discussion.

First, metric comparability remains limited. Studies report diverse performance indicators—such as classification accuracy, mean average precision (mAP), prediction fit (R^2^), and culturability (%)—across different organisms, datasets, and laboratory protocols. Direct comparison of headline numbers (e.g., “93% accuracy” vs. “89% accuracy”) is therefore often misleading without considering the organism scope, sample size, baseline method, and evaluation protocol used in each study.

Second, dataset heterogeneity and the scarcity of negative results impede model development. Most published cultivation datasets report only successful outcomes, while failed cultivation attempts—equally informative for ML—are rarely documented. Furthermore, standardized cultivation datasets analogous to ImageNet in computer vision do not yet exist in microbiology. However recent efforts such as the AGAR dataset [34] and the MediaMatch database [45] represent essential steps.

Third, cross-laboratory reproducibility has received insufficient attention. The vast majority of reported results come from single-laboratory settings. Factors such as medium batch variation, incubator calibration, operator technique, and local microbial contamination can substantially affect cultivation outcomes, yet few studies assess generalization across sites. Future benchmarking efforts should prioritize multi-site validation protocols.

Fourth, the distinction between computational prediction and experimental validation deserves emphasis. High identification accuracy (e.g., from Raman spectroscopy or colony imaging) does not automatically translate into successful isolation or cultivation outcomes. Identification outputs must be integrated with downstream processes—including target cell sorting, medium selection, and incubation parameter optimization—before culture recovery can be achieved. We encourage future studies to report end-to-end success rates (from sample to pure culture) alongside component-level metrics.

To improve rigor, we recommend that future studies adopt minimum reporting standards: (a) explicit description of baseline/control methods, (b) organism diversity and sample size, (c) whether validation is internal or external, (d) computational environment and reproducibility information, and (e) clearly separated reporting of identification accuracy versus cultivation success rate.

### 7.3. Future Directions

Facing these challenges, future AI-empowered microbial resource discovery will develop along the following directions:

• Microbial Foundation Models—Large-scale pre-trained models (i.e., DL models pre-trained on broad datasets and fine-tuned for specific downstream tasks, analogous to GPT in natural language processing) will be trained on massive microbial multi-omics data to learn universal microbial feature representations and biological principles;
• Digital Twin Microbial Factories—Constructing virtual simulation models (i.e., computational replicas that mirror real-time biological processes) of microbial cultivation processes to achieve “digital twins” from cells to bioreactors and from single strains to communities;
• Federated Learning and Data Sharing—Integrating microbial cultivation data from different laboratories globally while protecting data privacy;
• Physics-Informed Neural Networks (PINNs)—Embedding fundamental physicochemical laws and biological mechanisms into neural network architectures. For example, PINNs could encode Monod growth kinetics or thermodynamic constraints directly into network loss functions, ensuring that predictions of fermentation outcomes in food microbiology or wastewater treatment remain physically plausible even when training data are limited;
• Single-Cell Multi-omics and Spatial Microbiome AI—Combining single-cell sequencing, spatial transcriptomics, and other technologies to parse single-cell level heterogeneity;
• Interpretable AI and Causal Inference—Developing more advanced interpretable AI methods combined with causal inference [30];
• Automated and Autonomous Laboratories—Building highly automated intelligent laboratories to achieve full-process automation from sample preprocessing, cultivation, monitoring, and analysis to decision-making.

## 8. Conclusions

This review comprehensively examined the development of AI technologies in microbial isolation and cultivation over nearly three decades. The germination period (1997–2008) demonstrated the feasibility of AI for optimizing cultivation conditions [5,6,7,8,9,10] but was primarily limited to known cultivable microorganisms. The early exploration period (2008–2015) achieved systematic application of traditional ML methods [11], establishing a “data-driven” research paradigm. The rapid development period (2015–2019) demonstrated DL’s application potential in specific areas of microbiology [12,13,14,15,16,17,18,19], with breakthroughs in image recognition and gene expression analysis. The DL explosion period (2020–2022) established a multi-level technical framework spanning the genome, individual, and community levels [20,21,22,23,24], with cutting-edge technologies such as deep reinforcement learning [24] and interpretable AI [23] demonstrating unique advantages. The AI integration period (2023–present) achieved end-to-end intelligence and autonomous optimization capabilities [25,26,27,28,29,30,31,32,33,34,35,36,37,38,39,40,41,42,43,44,45,46,47,48,49,50,51,52,53,54,55,56,57], with landmark breakthroughs including gene function annotation tools that increased the number of verified transcription factors in *E. coli* to 276 [30]; LexicMap enabling rapid searching of millions of genomes [31]; intelligent carbon-nitrogen source prediction [32] achieving direct prediction from genomes to cultivation conditions; single-cell spectroscopic rapid identification [35,36,37,39] reaching minute-level speed; autonomous learning systems [43,44] reducing experimental iterations by 50% and improving throughput by 100-fold; intelligent medium prediction [45] successfully cultivating 38% of “unculturable” species; and culturomics [57] enhancing culturability from 1% to 15–30%.

The core value of AI technologies lies in bridging the vast gaps in human knowledge through knowledge acquisition and transfer, capturing rapid changes in microbial phenotypes through real-time analysis and dynamic optimization, and efficiently searching complex parameter spaces through system-level optimization. Technological iterations continue to drive methodological transitions from experience-driven to data-driven, from single-objective to systematic, and from passive screening to active design. Looking forward, cutting-edge technologies, including cross-scale foundation models, causal inference methods, autonomous research systems, function-oriented screening, and open collaboration platforms, will further transform microbial resource discovery. The deep integration of AI technologies with microbiological research is expanding the scope of microbial resource discovery.

## Figures and Tables

**Table 1:** Representative AI Applications in Microbial Isolation and Cultivation.

Development Stage	Application Level	Technology Type	Year	Key Performance	Advantages and Limitations	Ref.
Germination Period (1997–2008)	Individual	Hybrid neural network/Neural network simulation	1997–2007	Accurate prediction under noise interference	Adv: Precise prediction in noisy environments; Lim: Only applicable to known cultivable microorganisms	[5,6,7,8,9]
	Individual	Artificial neural network (fatty acid profile analysis)	2008	Identification accuracy of 85–90%	Adv: Significantly outperforms traditional methods; Lim: Requires standardized sample preparation	[10]
Early Exploration Period (2008–2015)	Genome	Extreme learning machine	2012	Fast training speed, suitable for short sequences	Adv: Rapid identification of novel microbial taxa; Lim: Shallow model with limited generalization capability	[11]
	Genome	Denoising autoencoder (ADAGE)	2016	Integrated 900+ datasets, discovered new virulence factors	Adv: Automatic pattern discovery without manual annotation; Lim: Requires large amounts of public data	[12]
	Community	ML association analysis	2016	Analyzed 200+ fermentation samples, revealed community-chemistry correlations	Adv: Discovering potential microbiome-metabolite connections; Lim: Cannot determine causal relationships	[13]
Rapid Development Period (2015–2019)	Individual	Naive Bayes (DNA melting curve)	2017	Accuracy > 90%, detection time < 2 h	Adv: Rapid distinction of closely related species; Lim: Requires specialized high-resolution melting equipment	[14]
	Individual	Hybrid modeling	2017	Prediction error 5–8%, better than single methods	Adv: More accurate prediction combining mechanistic knowledge and data learning; Lim: Limited generalizability	[15]
	Individual	Random forest (interaction network inference)	2018	Only 5% known interactions needed to predict 80% unknown	Adv: Greatly reduces experimental workload; Lim: Depends on quality and coverage of existing interaction data	[16]
	Individual	Support vector machine/Deep neural network	2018	100× faster, accuracy > 95%	Adv: High-throughput automated screening; Lim: Requires large annotated image datasets	[17,18]
	Individual	ML optimization (metabolic engineering)	2018	Target yield improved > 10-fold	Adv: Efficient exploration of vast parameter spaces; Lim: Specific to particular strains and products	[19]
DL Explosion Period (2020–2022)	Genome	Deep convolutional neural network (DeepVirFinder)	2020	Virus identification accuracy 93–98%	Adv: Discovery of novel viruses without reference database; Lim: Reduced performance for short sequences	[20]
	Individual	ML optimization (whole-cell catalysis)	2020	Yield improved > 40%, selectivity > 95%	Adv: Excellent enantioselectivity; Lim: Only applicable to specific reaction systems	[21]
	Community	DL interaction prediction	2020	Prediction accuracy 84%, distinguishes competition, symbiosis, etc.	Adv: Automatic learning of interaction patterns; Lim: Requires spatiotemporal pattern data	[22]
	Community	Interpretable AI (Predomics)	2020	Validated on 100+ datasets	Adv: Transparent and highly interpretable; Lim: Limited complex prediction capability	[23]
	Individual	Deep reinforcement learning (co-culture control)	2020	Automatic learning within 24 h	Adv: Suitable for difficult-to-cultivate microorganisms; Lim: Requires real-time sensors	[24]
	Genome	DL metagenomic tools	2021–2022	Automated viral binning, bacteriophage identification	Adv: Efficient processing of large-scale data; Lim: High computational resource requirements	[25,26,27]
AI Integration Period (2023–Present)	Genome	Constraint-based metabolic model	2023	Complete metabolic model constructed	Adv: Multiscale prediction guiding cultivation; Lim: Model construction is time-consuming	[28]
	Genome	Ultra-deep metagenomics	2025	756 ARG subtypes, 183 HGT events identified	Adv: Baseline for intrinsic resistance; Lim: Remote sampling	[29]
	Genome	DL gene function annotation	2026	Predicted ~5000 enzyme functions, 276 transcription factors verified	Adv: Attention mechanisms reveal molecular basis of predictions; Lim: Computational predictions require experimental validation	[30]
	Genome	LexicMap sequence alignment	2025	72× faster than BLASTn, only 7 GB memory	Adv: Rapid search of 2.3 million genomes; Lim: Index files require large storage	[31]
	Genome	Random forest (carbon/nitrogen source prediction)	2025	87% accuracy predicting 214 carbon and 95 nitrogen sources	Adv: Direct prediction of cultivation conditions without experimentation; Lim: Prediction quality depends on protein sequence quality	[32]
	Individual	YOLOv8 colony detection	2023	mAP > 92%, real-time detection	Adv: Fast speed; Lim: Difficulty recognizing complex colony morphologies	[33]
	Individual	AGAR standard dataset	2025	18,000 images with 336,442 annotated colonies	Adv: Unified benchmark for detection algorithm evaluation; Lim: Limited species coverage	[34]
	Individual	Raman spectroscopy + DL	2023–2025	Accuracy 89–95%, detection time < 5 min	Adv: Cultivation-free, single-cell level, low cost; Lim: Requires specialized equipment	[35,36,37,38,39]
	Individual	Deep transfer learning + hyperspectral	2024	Multimodal spectral information fusion	Adv: Non-destructive rapid detection; Lim: Hyperspectral equipment is expensive	[40]
	Individual	Microfluidics + ML	2024–2025	High-throughput screening	Adv: Single-cell resolution; Lim: Complex integration, difficult operation	[41,42]
	Individual	Active learning (BacterAI)	2023	~50% reduction in experimental iterations	Adv: Autonomous design without prior knowledge; Lim: Single experiment cycle remains bottleneck	[43]
	Individual	High-throughput culturomics	2023	100× throughput improvement, >10,000 new strains isolated	Adv: Discovery of new species; Lim: High equipment costs	[44]
	Individual	XGBoost medium prediction (MediaMatch)	2025	Successfully cultivated 38% of “unculturable” species	Adv: 40× efficiency improvement; Lim: Depends on historical cultivation data	[45]
	Individual	Long-term enrichment + single-cell sorting	2024	First pure culture of complete denitrifying methanotroph	Adv: Breakthrough for “unculturable” anaerobes; Lim: Extended cultivation time	[46,47,48]
	Community	Neural ordinary differential equations (mNODE)	2023	Prediction accuracy 75%	Adv: High interpretability; Lim: High computational complexity	[49]
	Community	Multi-omics + co-cultivation validation	2023	Mutualism 31–45% via cobalamin sharing	Adv: Guides helper species selection; Lim: Environment-specific	[50]
	Community	Graph neural network (microbe-disease/interaction prediction)	2023–2024	Prediction accuracy > 90%	Adv: Integration of heterogeneous information; Lim: Requires high-quality knowledge graphs	[51,52,53,54]
	Community	Data-driven colonization prediction	2024	No kinetic model required	Adv: Direct prediction of colonization outcomes; Lim: Generalization capability needs validation	[55]
	Community	Flow cytometry + quantitative analysis	2025	Accurate quantification of species ratios in mock communities	Adv: Rapid quantification of community composition; Lim: Challenges in real co-culture systems	[56]
	Community	Multidimensional culturomics	2025	Culturability improved to 15–30%	Adv: Systematic integration of multiple technologies; Lim: Requires significant equipment and personnel investment	[57]

Note: Performance metrics are reported as published in the original studies and may not be directly comparable across entries due to differences in organism scope, dataset size, evaluation protocol, and laboratory conditions. Adv = Advantages; Lim = Limitations.

## Data Availability

No new data were created or analyzed in this study. Data sharing is not applicable to this article.
